# Ethical issues with geographical variations in the provision of health care services

**DOI:** 10.1186/s12910-022-00869-7

**Published:** 2022-12-06

**Authors:** Bjørn Hofmann

**Affiliations:** 1 Institute for the Health Sciences, The Norwegian University of Science and Technology (NTNU), PO Box 191, 2801 Gjøvik, Norway; 2 The Centre for Medical Ethics, University of Oslo, PO Box 1130, 0318 Oslo, Norway

**Keywords:** Geographical variation, Justice, Harm, Beneficence, Autonomy

## Abstract

Geographical variations are documented for a wide range of health care services. As many such variations cannot be explained by demographical or epidemiological differences, they are problematic with respect to distributive justice, quality of care, and health policy. Despite much attention, geographical variations prevail. One reason for this can be that the ethical issues of geographical variations are rarely addressed explicitly. Accordingly, the objective of this article is to analyse the ethical aspects of geographical variations in the provision of health services. Applying a principlist approach the article identifies and addresses four specific ethical issues: injustice, harm, lack of beneficence, and paternalism. Then it investigates the normative leap from the description of geographical variations to the prescription of right care. Lastly, the article argues that professional approaches such as developing guidelines, checklists, appropriateness criteria, and standards of care are important measures when addressing geographical variations, but that such efforts should be accompanied and supported by ethical analysis. Hence, geographical variations are not only a healthcare provision, management, or a policy making problem, but an ethical one. Addressing the ethical issues with geographical variations is key for handling this crucial problem in the provision of health services.

## Background

Geographical variations in health services have been documented in a wide range of fields, such as access to care [1–6], standards or quality of care [7], drug use [8, 9] and surgery [10–14], service provision or organization [15–21], application and quality of diagnoses [22–24], tests used in primary care [25], medical imaging [26–32], number of referrals [33] and treatments [12, 13, 34–39], as well as treatment outcomes [40–42].

Globally it is well documented that healthcare needs are higher among the poor, while access to health care and healthcare use is higher among the rich [43–47]. More locally it is known that the health services placements are not attuned the groups with the highest need [48, 49].

Moreover, geographical variations in the allocation of resources for and the provision of health care [50, 51] as well as for the access and utilization of health services [52–59] have been documented and discussed.

Many countries have published atlases of geographical variations for a wide range of services Following Jack Wennberg’s seminal book *Tracking Medicine* [60]. The Dartmouth Atlas has inspired a wide range of similar work in many countries, such as in the NHS in UK [61]. See Table 1.

**Table 1:** Examples of atlases for geographical variations of health services

Name	Description	Reference/link
The Dartmouth Atlas of Health Care	Extensive set of (interactive) atlases of US health services	https://www.dartmouthatlas.org/
Australian Atlas of Healthcare Variation	Encompassing (interactive) atlases for selected topics 2015, 2017, 2018, 2021	https://www.safetyandquality.gov.au/publications-and-resources/australian-atlas-healthcare-variation-series
NHS Atlas of Variation in Healthcare	Atlases for a range of fields from 2012 till 2020	https://www.england.nhs.uk/rightcare/products/atlas/
New Zealand Atlas of Healthcare Variation	A wide range of atlases organized by domains	https://www.hqsc.govt.nz/our-programmes/health-quality-evaluation/projects/atlas-of-healthcare-variation/
Scottish Atlas of Healthcare Variation	Interactive map for selected areas from 2013 to 2019	https://www.isdscotland.org/products-and-services/scottish-atlas-of-variation/introduction/
Institute for Clinical Evaluative Sciences research atlases	Atlases covering a range of system-related and disease-specific topics, from 1994 till 2020	https://www.ices.on.ca/Publications/Atlases-and-Reports
Atlas des variations de pratiques médicales	Atlas for variations in medical services (surgery) 2016	https://www.irdes.fr/recherche/ouvrages/002-atlas-des-variations-de-pratiques-medicales-recours-a-dix-interventions-chirurgicales.pdf
German atlases over health care provision	Several atlases of a wide range of services provided by various institutions.	https://www.versorgungsatlas.de/ http://www.kbv.de/html/gesundheitsdaten.php http://www.gesundheitsatlas-bw.de https://www.lgl.bayern.de/gesundheit/gesundheitsberichterstattung/gesundheitsatlas/ia_report/atlas.html
Swiss Atlas of Health Services	Altlases of 30 specific services	https://www.versorgungsatlas.ch/index.php/de/versorgungsatlas/
Norwegian Health Atlas	Atlases over specific types of health services	https://helseatlas.no/en

Many such well-documented geographical variations cannot be explained by demographical differences or in epidemiological terms and appear to be iatrogenic or systemic. Accordingly, it has been claimed that such geographical variations indicate underuse and overuse [62–71] and pose challenges to clinical practice, health services provision, and health policy making. This has spurred a series of initiatives, such as the Choosing Wisely Campaign, Too Much Medicine (BMJ) series, Smarter Medicine movement, Prudent Health Care, Slow Medicine, Do Not Do (NICE), just to mention a few [72].

However, despite much attention and many measures to reduce geographical variations in health care services, the problem prevails. One of the reasons for this may be that little attention has been on the ethical aspects of geographical variations. To a large extent scholars have revealed the geographical variations and assumed that everyone understands why they are wrong. In fact, no comprehensive explicit ethical analysis of this has been performed. Accordingly, *the objective of this study is to analyse the ethical aspects of geographical variations in health services provision in order to guide health policy making*.

The article sets out to analyse geographical variations in terms of the four bioethical principles, justice, non-maleficence, beneficence, and autonomy [73]. In order to address the issue of overuse and underuse in geographical variations, the article then investigates the normative leap from the description of geographical variations to the prescription of right care, from *is* to *ought*. Finally, it addresses the question of whether the ethical issues are contingent on the type of health services. Based on these steps it concludes that acknowledging the ethical issues can help understanding why unwarranted geographical variations^1^ are bad and provide resources to mitigate the problem.

The reason that principlism is applied here is that it has been prominent within health care ethics since the 1970s [74, 75]. According to Beauchamp and Childress, the four principles are universally valid norms, and are synopsised as follows: “respect for autonomy (the obligation to respect the decision making capacities of autonomous persons); non-maleficence (the obligation to avoid causing harm); beneficence (obligations to provide benefits and to balance benefits against risks), and justice (obligations of fairness in the distribution of benefits and risks)” [73]. Despite its weaknesses, this principle-based approach has demonstrated to be applicable, sustainable, and highly prolific in a wide range of fields [76].

One important caveat is that this study addresses geographical variations that are not justified by relevant geographical differences. Clearly, geographical variations in the provision, access, and use of health services can be warranted due to geographical variations in the characteristics of the population, e.g., in age, severity or burden of disease, vulnerability etc. Hence, this study is not about geographical variations in the provision, access, and use of health care services in general, but only those that are not warranted by differences in populations, i.e., where there are no relevant moral differences. Unfortunately, as referred to above, there are very many such unwarranted geographical variations and addressing their ethical aspects is important to come to grips with the problem.

## Main text

### Justice

Geographical variations in health services that do not correspond to demographical or epidemiological differences are ethically challenging as they express unwarranted differences in care. As indicated in the introduction, this can be differences in the provision of care, access to care, use of health services, in quality and standard of care, in outcomes of care etc.—all infringing the principle of justice [23]. Some patients will get more, and some will get less, depending on where they live or are served. As pointed out in the introduction, there are socioeconomic, racial, ethnic, risk- and health-related differences in the provision, access, use, and quality of care [1–59] that are profoundly unjust.

As already pointed out, globally healthcare needs are higher among the poor, while access to health care and healthcare use is higher among the rich [43–47]. Locally health services are not placed where the needs are highest [48, 49].

Unwarranted geographical variations express variation in resource use [25], inefficient health services management [25], overuse, e.g., in terms of unnecessary surgery [77], but also of underuse. Overuse of low-value care represents an opportunity cost, reducing the access to efficient care for others. Low-value care is defined as “an intervention in which evidence suggest it confers not or very little benefit for patients, or risk of harm exceeds probable benefit or, more broadly, the added costs of the intervention do not provide proportional added benefits” [78].

Correspondingly, geographical variations raise issues of equity for a wide range of health services [2, 6, 43, 44, 46, 47, 51, 59, 79, 80] and pose challenges with respect to balancing concerns for equity with concerns for efficiency. As pointed out by Rice and colleagues, there exists a trade-off “between equity and efficiency in the allocation of health care resources. The pursuit of efficiency, in the sense of equalising the marginal cost of a unit of health gain, must be moderated by equity considerations” [50].

The main point ethically is that geographical variations in health services (that do not stem from morally relevant differences) are challenging, as they result in unwarranted discrimination, inequity, and injustice. While the causes for these geographical variations are well-known, e.g., structural, socioeconomic, and racial/ethnic differences, biases, stigmatization, as well as differences in living conditions, climate and power structures, the solutions are difficult to obtain exactly due to these ingrained causes. The important lesson is that while some of the causes of and solutions for geographical variations in the health services can be found within the services (see below) many of them have to be found outside the services.

Hence, unwarranted geographical variations in the health services infringes the principle of justice and thereby poses a substantial problem to health policy makers, health care managers, health professionals, and patients.

### Harm

Another ethical challenge with geographical variations in the health services is that they are indicators of harm. There are two main forms of harm related to such variations: harm from underuse and harms from overuse.

Harm from underuse is increased morbidity and mortality due to late or lack of provision, access to, or use of health services. A patient who could have been diagnosed and successfully treated for cancer (at an earlier stage), but who suffers more as the result of the delay or neglect, is a typical example. The consequence of underuse is poor health outcomes in terms of welfare and health shortfall [59]. Underuse has been documented in a wide range of health services [81]. The underuse of each of these services has a range of specific harms, that should be addressed in each specific case.

For overuse, there is a plethora of harms. The harms of *diagnostic overuse* include false positive test results (anxiety and follow up), incidental findings of little or no significance (incidentalomas), overdiagnosis (diagnosis of a condition that would never harm the person) [82], side effects of diagnostics (e.g., reactions to contrast media or ionizing radiation in imaging), unnecessary subsequent diagnostics (to verify findings), and unnecessary subsequent treatment (overtreatment). Harms from *therapeutic overuse* include direct (expected) harms, adverse side-effects, and complications from treatments. Overuse has been documented for a wide range of health services globally [62, 83]. The overuse of each of these services has a range of specific harms, that ought to be addressed in each specific case.

Hence, unexplained geographical variations are ethically problematic as they indicate specific harms from underuse and overuse. Each of these harms need to be addressed specifically when investigating and tackling geographical variations.

### Beneficence

Another key challenge with geographical variations is that it expresses variation in quality of care [84]. Geographical differences in the quality of health services can result in worse outcomes for patients depending on where they live (or are served) and not on their health condition or characteristics [13, 16, 35, 38]. This goes for both benefits and harms (safety, as discussed above) and may infringe the principle of beneficence, as the geographical variations indicate that certain services are not of best benefit to patients.

One must also ask whether the same goes for those getting better care than others? It can be argued that too much of services with a benefit-to-harm-ratio > 1 cannot be bad. For example, to provide an effective treatment that is above the cost-effectiveness threshold [85] will benefit the patients getting the treatment. Hence, extra use (“overuse”) of such treatments is good (according to the argument). However, as pointed out above, providing (cost) effective services may generate injustice due to the opportunity cost (infringing the principle of justice).

Correspondingly, getting less of a service with a benefit-to-harm-ratio < 1 than others is good from the perspective of the principle of beneficence. Again, other principles, such as the principle of justice, kicks in to differentiate the cases.

Another interesting and relevant issue is when services have other effects than intended, making them beneficial in some other (sometimes undocumented) way, and that this beneficence is acknowledged and applied differently geographically. One example of this is the anxiolytic effects of radiological services. Many radiological investigations are not used because the health professionals believe that patients have specific pathologies (i.e., the pre-test-probability is low), but because the examination will have a reassuring or relaxing effect on the patient or in order to do something (*ut aliquid fiat*) [86, 87]. If the acknowledgement and application of such anxiolytic (or other beneficial) effects are unevenly distributed, it can be argued that geographical variations are good, or at least not bad. One problem, however, is that many such effects are uncertain and hard to document [88]. Hence, the benefit is unclear. Additionally, we encounter problems with the principle of justice, discussed above.

Thus, geographical variations in the provision, access to, and use of health services represent differences in benefit to patients, and thereby violate the principle of beneficence. While having more of a beneficent service or having less of a non-beneficent service can be considered a good, this may infringe other principles, such as justice.

### Respect for autonomy

When it comes to the principle of respect for autonomy, one can differentiate between variation in the experience and execution of patient autonomy and variations in professionals’ respect of patients’ autonomy. Moreover, one may distinguish between variation in autonomy due to variations in the provision, access, and use of health services and due to variations in the premises for executing autonomy, e.g., provided information. However, such distinctions are not clear.

Geographical variations in the execution of patients’ autonomy have been documented [89, 90], e.g., due to socioeconomic differences. Patient autonomy is also considered by health professionals to be a driver of unnecessary care, such as low-value radiological examinations [91], and as far as patient demand and professional integrity have geographical variations, this can cause geographical variations in the execution of patients’ autonomy.

Correspondingly, there are geographical variations due to differences in the respect for patients’ autonomy, e.g., in withdrawal and limitation of life-sustaining treatments for children [90]. Such variations can be due to personal professional preferences [92].

Yet another way that geographical variations can be challenging considering the principle of respect for autonomy is that core criteria for making autonomous choices are not met. While geographical variations in the capacity to consent can warrant geographical variations in autonomy, variations in information (disclosure) and voluntariness may not. As professional preferences are shown to differ from and influence patients’ decision-making [93], geographical variations in the provision and use of health services can be due to differences in information and voluntariness.

As geographical variations unfairly affect low income, vulnerable, and underprivileged groups, they will depend on charity and are subject to another type of paternalism, as the few “lucky persons” who are offered “extra services” have little or no choice (i.e., lack voluntariness) and are not well informed.

This is not the place to enter a detailed discussion on the drivers of differences in respect for patients’ autonomy in health care. The point here is that geographical variations due to different levels of paternalism are ethically problematic. Hence, when addressing geographical variations, we should address the issue of paternalism.

### From geographical variations to right care

As referred to in the introduction, there are many reports of geographical variations of health services, and it is generally assumed that such variations are ethically problematic, as they represent underuse and overuse. However, one thing is to describe geographical variations and to assume underuse and overuse. Quite another thing is to decide what is too little and too much, i.e., what is the right level of care. Defining the standard of care is challenging: “A further major lacuna in seeking to make ethical policies operational has been the reluctance to specify what constitutes a “standard” package of health care” [50]. In general, effective care gives indication of “right care” [94].

Geographical variations have been reported for decades [95] without there been significant changes [96]. One reason for this may be the difficult move from the description of geographical variations to the prescription of the right level and quality of care.

Many reports and atlases (e.g., in Table 1) present medians or mean for the various types of geographical variations, and thereby implicitly infer about under- and overuse based on such measures. However, this approach is by far justified, as it mixes quantity and quality and implies reasoning from *is* to *ought*.

To address such issues a wide range of professional and national bodies have established Clinical Care Standards [97] and National Safety and Quality Health Service Standards [98]. Guidelines, checklists, and norms for right care are elaborated. See for example https://www.england.nhs.uk/rightcare/. In radiology an evidence- and consensus-based set of appropriateness criteria has been developed [85]. While these approaches are powerful tools to reduce unwarranted geographical variations in health services, they raise ethical issues themselves. How do we know and decide what is appropriate?

In the Lancet series on this issue, *right care* is defined in terms of the four ethical principles: “What is right care? In its simplest definition it is care that weighs up benefits and harms, is patient-centred (taking individual circumstances, values, and wishes into account), and is informed by evidence, including cost-effectiveness” [99]. To make this practically applicable two important questions raise: (1) Which outcome measures should be used to assess good or appropriate care? (2) Where do we set the limits for good or appropriate care (for the selected outcomes)? For example, when deciding on appropriate imaging, which outcome do we apply? Do we use subsequent improved health, changed subsequent health management (diagnostic or therapeutic path), reduced number of negative tests, reduced number of false (negative or positive) test results etc? If we decide to measure outcome in terms of changed subsequent health management, the question becomes how much this has to be changed in order to be good care. Where to set the limit very much depends on the context (local, national, global). Hence, the first question hinges on the value base of health care [100–103] and the second on the line-drawing problem in medicine [104, 105]. Both questions are ethically relevant.

Hence, unwarranted geographical variations of health services pose the core question “*what is right care?*” which is an ethically relevant issue. While professionally developed standards, guidelines, and checklists can be helpful for deciding on “right care” in specific contexts [106] there is empirical evidence that much more work needs to be done in order to reduce geographical variations. In particular, defining good care more explicitly is a crucial task which requires ethical reflection.

The point here is that paying attention to the ethical issues with geographical variations can make valuable contributions. In particular, addressing the injustice and potential harm that geographical variations entail, the lack of beneficence that they imply, and the paternalism that they indicate can help adjusting care. Knowing why geographical variations are wrong can help mitigating them.

As geographical variations may be contingent on the type of health service, let me briefly address this issue explicitly.

### Geographical variations contingent on type of service

According to one of the pioneers in the studies of practice variations, Albert Mulley, “[i]f all practice variation were bad, then it would be an easy problem to solve” [94]. Some variations are good, and some are bad, and the type of service may be relevant for discerning between them.

Health services are frequently divided into necessary services, preference-sensitive services, and supply-driven services [73]. *Necessary services* are services that are documented to be crucial to people’s health and/or are strongly recommended in guidelines or standards of care, while *preference-sensitive* care are “those interventions in which there is a choice between at least two treatments that have different risks and benefits” [84], and *supply-sensitive services* are directed by the capacity or the interests of the local health care system and not by patients’ needs or preferences.

Accordingly, it may be argued that variations in preference-sensitive services are less ethically problematic than in necessary services. Certainly, variations in preference-sensitive services may not be ethically challenging as they can express variations in people’s preferences. However, if there are great geographical variations in preference-sensitive services in homogenous populations, this warrants attention as the variations then do not stem from patients’ preferences but can be iatrogenic or systemic (and thereby threaten the respect for autonomy).

Conversely, geographical variations in necessary services appear ethically challenging, as this indicates unwarranted differences in access or quality of care. However, there are not always agreements on what are necessary services, and there may be (inter)national differences of what is standard of care. Again, if there are geographical variations in necessary services in professionally and demographically homogeneous areas, this can be ethically problematic, especially with respect to differences in access to and quality of care. This poses challenges with respect to beneficence and non-maleficence.

When it comes to supply-sensitive services, it has been argued that these are ethically challenging as such, as they are (a) independent of patients’ preferences and (b) are unnecessary services [60, 84]. Accordingly, these can cause problems with patient autonomy and justice (opportunity cost, see below).

Hence, although there may be reasons to believe that geographical variations in necessary services are more problematic than in preference-sensitive services, and that they are always challenging in supply-sensitive services, geographical variations can pose ethical problems in all types of services. Moreover, geographical variations in necessary services may be more pronounced in low-income and vulnerable groups posing challenges with beneficence and justice, but also with non-maleficence and paternalism.

## Discussion

This study has focused on geographical variations. There are of course many other types of variations that are ethically relevant, e.g., racial and gendered variations. However, these warrant separate studies. When geographical variations occur in homogeneous populations, they represent a type of ethical challenges that are of general interest, and that can be relevant also for more specific types of variations.

There are also ethical aspects of geographical variations in clinical research [107] and there are ethical challenges with the design of studies of geographical variations [108]. This raises a series of issues that are beyond the scope of this study. Again, geographical variations in specific fields warrant separate studies.

There are of course also many other ethical challenges with the health services beyond having unwarranted geographical variations, and there are many reasons for and drivers of geographical variations that have ethical issues. Addressing these as well would embellish the extension and content of this study beyond limits.

As indicated, overuse and underuse are potential ethical problems with geographical variations is. However, there are many conceptions of overuse and underuse. I have pointed to the problem of deciding on (appropriate) outcomes for assessing under- and overuse and on the line-drawing problem. Addressing the problem of under- and overuse in general is important but beyond the scope of this study.

Yet another limitation is the ethical framework applied here. Principlism is widely criticized and has its weaknesses [14, 109–111]. Other perspectives than principlism are relevant [112] and other principles are pertinent. Solidarity and sustainability are but two examples [113, 114]. Principles for priority setting, such as severity or need, effectiveness, equity, and cost(-effectiveness) are also relevant. However, needs, severity, and effectiveness are partly covered by the principle of beneficence, and cost-effectiveness and equity are mostly covered by justice. Certainly, the analysis of geographical variations in the light of such (other) principles can add value to this study and is encouraged. However, the principles of biomedical ethics are well established and principlism has served as a fruitful framework for a wide range of studies in health care ethics.

Moreover, the principles are used to analyse geographical variations in general. Hence it provides a generic analysis. For the geographical variations of specific health services one can make more in-depth analyses of the principles. One example of this is a study of geographical variation in compulsory hospitalisation where the framework of this article has been applied for an in-depth analysis [115]. One may also argue some principles are more relevant for addressing the ethical issues with geographical variations, as the provided analysis puts more emphasis on some principles (beneficence and justice) than others (non-maleficence and autonomy). While this may be correct for the generic analysis, this does not have to be so for analyses of specific health services. See for example [115].

As pointed out in the introduction, geographical variations can be justified by (known or unknown) differences in individuals, groups, or populations. Adjustments of age, gender, socioeconomic, and other factors cannot rule out profound but ignored geographical differences. While this is an interesting (principled and methodological) objection, this study has presumed that there exist geographical variations that are not based on such differences. Unfortunately, it is rarely the case that geographic variation of health care services reflects demographic or epidemiological differences even in high income countries. On the contrary, a range of differences in health risk, socioeconomic status, and race are not reflected in the provision, access, and use of health care. Rather where the needs are highest, the provision, access, and use of health care is lowest. Hence, there is a “double injustice” when those who need it the most have the least.

Thus, while variation can be seen as a good in general, e.g., with populational diversity, this study is on a topic where there are good arguments (presented above) that variation is not an intrinsic value.

Nonetheless, it can be argued that geographical variations are acceptable in areas where “right care” is vaguely defined or covers a broad spectrum of services. Certainly, this is a valid argument as then differences may not matter. However, many of the variations that are documented in the literature (see above) are very large and go beyond variations within “right care” or “acceptable care.”

Moreover, paying attention to the ethical problems with geographical variations can motivate measures to reduce unwarranted variations. Documenting and illustrating what is wrong with geographical variations can direct us towards better care. Accordingly, we must show how the variations represent injustice in terms of discrimination of specific groups, e.g., by disclosing detailed opportunity costs. We must demonstrate how specific persons and groups are harmed rather than helped. Additionally, we must reveal the lack of respect for autonomy when people are not informed about the quality and safety of services as part of geographical variations.

It is also important to notice that while people in specific places may have reduced access and use of health services, they still may not consider themselves to be disadvantaged [116]. Furthermore, this article has not been focused on the expression, extension, and causes of geographical variations, but on the ethical issues. While the former issues are well covered by the literature and reviewing this literature would be beyond the scope of this study, the latter has only partly been covered.

Last but not least, this study does not answer the question of whether geographical variations can be avoided. Neither have I provided a detailed framework for what we can do about geographical variations in health services [61]. More modestly, I have pointed to specific ethical challenges with geographical variations related to the four principles in bioethics, justice, non-maleficence, beneficence, and autonomy. I have also argued that addressing these issues can come together with other efforts to reduce unwarranted geographical variations. As other approaches, such as campaigns, guidelines, and standards of care have not been successful, addressing the ethical issues identified here may contribute to, support, and intensify such efforts. Success can of course not be guaranteed. However, as the ethics of geographical variations has gained so little attention, there are good reasons to hope that making them more explicit can enhance the efforts to mitigate the problem.

## Conclusion

The objective of this article has been to analyse the ethical aspects of geographical variations in the health services. The analysis of geographical variations in terms of the four bioethical principles revealed problems of injustice, harm, lack of beneficence, and paternalism. Lastly the study revealed that the description of geographical variations does not automatically define or result in better care. To come from the description of the problem to its solution requires that we address two basic normative problems: defining goodness of care and drawing the line between good and bad care. Although it appears plausible that geographical variations are more ethically challenging for necessary services than for preference-sensitive services, geographical variations can pose ethical challenges in all types of services, including supply-sensitive services. Various approaches with respect to developing policies, guidelines, checklists, and standards of care are helpful, but need to be supplemented with and supported by attention to the ethical issues analysed in this article. Showing explicitly how geographical variations pose injustice, harm, lack of beneficence, and paternalism can make a crucial contribution to addressing the problems with geographical variations in the health services.

## Data Availability

All data are available in publication.

## Footnotes

1 I define unwarranted geographical variations as variations that cannot be explained and justified by underlying variations in population characteristics or acceptable professional discretion.
